# Multicenter evaluation of the DEXTER® Robotic Surgery System for Total Hysterectomy with Adnexal Surgery

**DOI:** 10.1007/s11701-025-02647-0

**Published:** 2025-09-26

**Authors:** Marietta Gulz, Marie-Lucile Bodet, Matthias Streich, Anna Habegger, Martin Heubner, Damaris Erhardt, Borjana Bogdanovic, Michael David Mueller

**Affiliations:** 1 Department of Obstetrics and Gynecology, Bern University Hospital, 3010 Bern, Switzerland; 2 Department of Gynecology, Groupe Hospitalier Saintes – Saint-Jean-D’Angély, Saintes, France; 3 Department of Gynecology and Obstetrics, Spital Interlaken, Spitäler Fmi AG, Interlaken, Switzerland; 4 Department of Gynecology, Kantonsspital Baden AG, Baden, Switzerland; 5 Distalmotion SA, Epalinges, Switzerland

**Keywords:** DEXTER robotic surgery system, Hysterectomy, Minimally invasive surgery, Robotic-assisted surgery

## Abstract

Minimally invasive hysterectomy is the preferred surgical approach for benign gynecologic conditions. The DEXTER® Robotic Surgery System was developed to address the limitations of conventional robotic systems, offering a mobile, modular, and accessible alternative adaptable to various surgical environments. This prospective, multicenter, single-arm study evaluated the clinical performance and early postoperative safety of the robotic-assisted hysterectomy with adnexal surgery for benign indications with DEXTER. The primary endpoints were short-term safety, assessed by the occurrence of Clavien–Dindo grade ≥ III adverse events up to 42 days postoperatively, and procedural success without conversion to laparoscopy or open surgery. Fifty-two patients were enrolled across four European centers—one university hospital and three community hospitals. All procedures were successfully completed without intraoperative complications or conversions. The mean total skin-to-skin operative time was 121.9 ± 42.7 min, and the mean estimated blood loss was 87.8 ± 93.8 mL. The system also demonstrated efficient setup with a mean docking time of 3.8 ± 1.3 min, as well as the feasibility of a three-trocar technique for minimal scarring and potential for outpatient application in select cases. The mean time to discharge was 2.0 ± 0.9 days. One major Clavien–Dindo IIIb complication occurred. No device-related adverse events were reported. These results confirm the safety and performance of DEXTER in robotic-assisted hysterectomy and support its role in expanding access to robotic-assisted surgery in diverse clinical settings.

*Trial registration*: ClinicalTrials.gov NCT06473675.

## Introduction

Minimally invasive hysterectomy is widely endorsed as the preferred surgical approach for benign gynecologic conditions. International societies consistently recommend laparoscopic hysterectomy as the first-line option when vaginal surgery is not feasible [1–3]. Recent position statements further support the feasibility and safety of robotic-assisted hysterectomy (RAH), reporting outcomes comparable to those of conventional laparoscopy [4, 5].

RAH offers clear advantages in terms of precision, ergonomics, and shorter hospital stays, making it an attractive option for both patients and surgeons [6]. Nevertheless, access to RAH remains limited. Most existing robotic platforms require dedicated operating room (OR) space, institutional resources for surgeon training, and substantial infrastructure. These requirements pose significant barriers for outpatient care settings, including ambulatory surgery centers, which healthcare systems are increasingly shifting towards [7, 8]. These limitations reduce introduction and scalability of robotic surgery in precisely the outpatient settings where minimally invasive techniques are most encouraged, particularly for benign indications. Thus, there is growing demand for robotic solutions that fit into outpatient sites of care and offer flexibility, efficiency, and cost-effectiveness without compromising the ability to operate precisely and minimally invasively in tight anatomical spaces.

The DEXTER® Robotic Surgery System (Distalmotion SA, Epalinges, Switzerland), granted CE marking for gynecologic, urologic, and general surgery and introduced to the market in 2022 [9], is a small, mobile platform designed to integrate into existing surgical environments, without the need for dedicated infrastructure [10–12]. Its open architecture enables the use of preferred visualization systems and laparoscopic electrosurgical generators, making it adaptable to a range of surgical settings [13]. Recently approved in the United States, DEXTER has demonstrated early value in ambulatory procedures for inguinal hernia repair [14] and cholecystectomy, and its adoption is expanding across outpatient centers in both Europe and the United States.

However, prospective clinical data on its use in gynecologic surgery remain limited. This prospective multicenter study aimed to evaluate the clinical performance and early postoperative safety of DEXTER in routine RAH with adnexal surgery for benign indications.

## Materials and methods

### Study design and setting

This was a prospective, multicenter, single-arm clinical investigation, conducted to generate clinical evidence to expand the regulatory clearance of DEXTER in the United States to include hysterectomy. The study was registered under ClinicalTrials.gov identifier NCT06473675 and approved by the Ethics Committees according to local and national regulations of the participating countries. The study was performed in accordance with the principles of the Declaration of Helsinki, Good Clinical Practice (GCP), the European Regulation on medical devices 2017/745, ISO 14155:2020, and 21 CFR 812.28. All subjects provided informed consent and underwent RAH with adnexal surgery using DEXTER.

Four clinical centers in Switzerland and France participated in the study: one university hospital and three community hospitals. Reflecting real-world clinical conditions, the study involved five primary operating surgeons across the participating sites: three were robotically naïve at study initiation, one had fewer than 5 years of robotic surgery experience, and one was an expert robotic surgeon with over 10 years of experience with the da Vinci system.

This study had two primary objectives: to confirm the safety of RAH with adnexal surgery using DEXTER by estimating the rate of Clavien–Dindo grade III–V adverse events up to 42 days postoperatively, and to assess the performance of DEXTER by estimating the rate of successful procedure completion without conversion to another surgical approach. Secondary objectives included the evaluation of additional intra- and postoperative outcomes. Safety outcomes comprised the intraoperative complications, all postoperative complications up to 42 days (Clavien–Dindo classification), estimated blood loss (in milliliters), rehospitalization, and mortality. Performance outcomes included docking, robotic console and total (skin-to-skin) operative times (in minutes), and the use of additional trocars beyond the three used for endoscope and two robotic arms. The study also assessed the length of postoperative hospital stay (in days) and revision surgery rates.

### Patient selection criteria

The study included adult patients scheduled to undergo elective robotic-assisted or laparoscopic hysterectomy with adnexal surgery for benign indications. Inclusion criteria required the ability to provide informed consent and attend a 6-week follow-up appointment. Patients were followed for 42 days postoperatively, to adequately assess vaginal cuff healing and other safety parameters.

Exclusion criteria included morbid obesity (BMI > 40), any contraindications to endoscopic surgery, bleeding diathesis, pregnancy, implanted cardiac devices, planned concomitant procedures beyond adnexal surgery, legal incapacitation, and participation in another interventional trial. Procedure-specific exclusions comprised diagnosed malignancy of reproductive organs, prior major abdominal or pelvic surgery, and uterine fibroids larger than 10 cm. Major surgery was defined as any previous procedure (excluding cesarean section) involving a large abdominal incision (> 10 cm) and/or extensive resection altering normal anatomy.

### Use of the DEXTER Robotic Surgery System

**Fig. 1:**
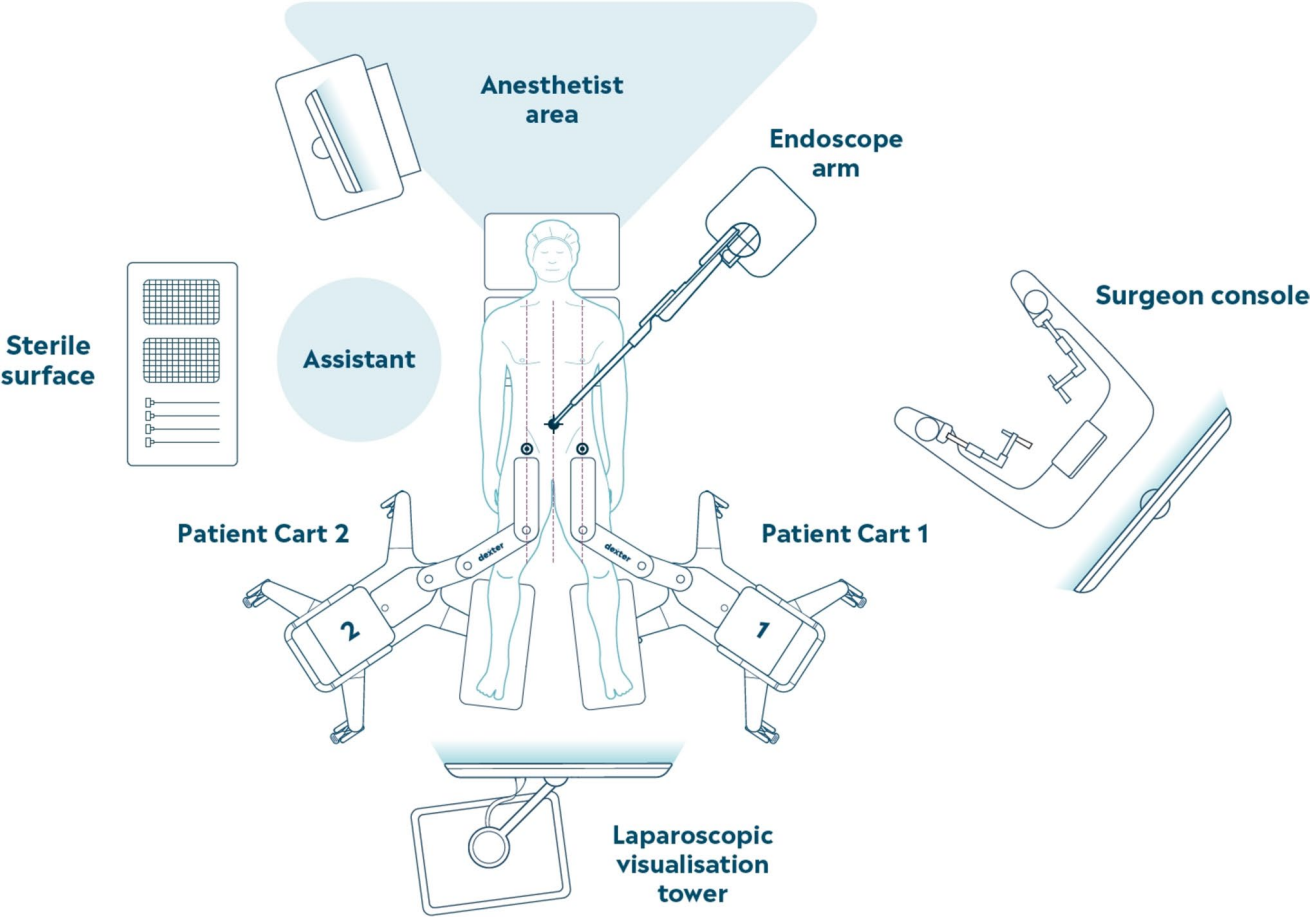
OR setup for RAH using DEXTER

In this study, DEXTER was used in a variety of hospital settings. Depending on the clinical site and OR size, the system was either already in the OR in its compact storage mode, or routinely transported into the OR before the procedure, enabled by its small, mobile footprint. The modular components of the system were positioned in each OR such that the two patient carts with robotic instrument arms approached the patient bed from the sides, the endoscope arm was positioned by the patient’s head, and the sterile surgeon console was positioned nearby, allowing free communication between the console surgeon and the OR staff at the patient bedside (Fig. 1). All of the components, including the surgeon console, were draped for sterility enabling the surgeon to remain sterile throughout the entire procedure. Considering DEXTER is compatible with standard laparoscopic tower equipment including visualization systems and energy devices, each clinical site used its existing setup (including Erbe Elektromedizin, Olympus, and Karl Storz systems).

**Fig. 2:**
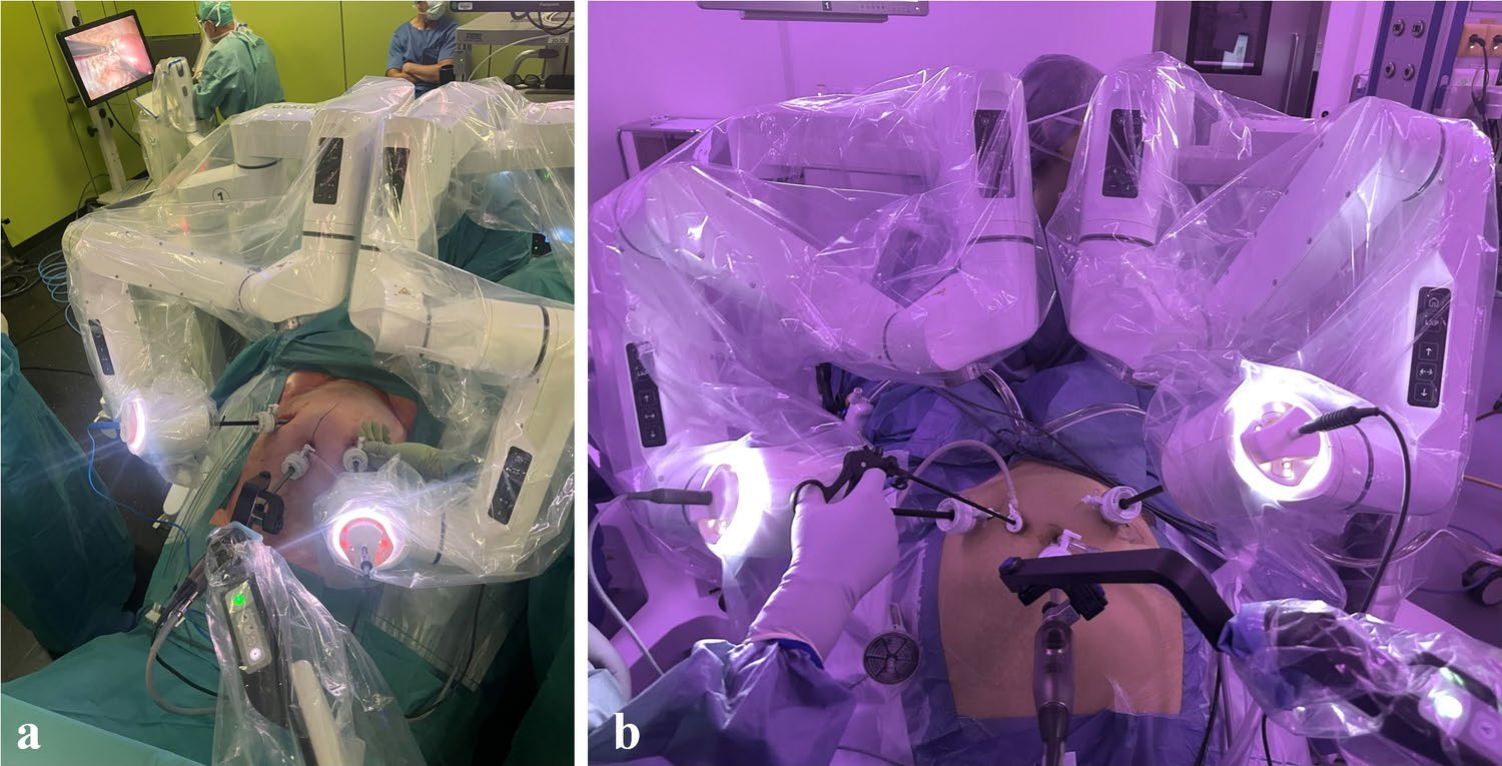
**a** Three-port setup for hysterectomy using DEXTER, **b** standard four-port setup including the three robotic arms with one additional assistant trocar

Previous studies have reported trocar setup for DEXTER use [10, 15]. In this study, the trocar setup consisted of three ports: one for the endoscope (10–12 mm in diameter) and two for robotic instruments (8–10 mm in diameter) (Fig. 2a). In general, the endoscope port was placed at the umbilicus. The instrument ports were positioned approximately two finger width medial to the anterior superior iliac spines on a diagonal toward the umbilicus, or 11 cm from the pubic symphysis. If needed, additional ancillary 5-mm trocars were used for the assistant (Fig. 2b) and placed in between the umbilical port and the left 8-mm port.

Following port placement and docking of the robotic system, the procedure began with adhesiolysis if required. The round ligaments were divided, and the uterus, fallopian tubes, and ovaries were mobilized according to the planned surgical approach. Dissection of the vesico-uterine space, coagulation and transection of the vascular pedicles, and colpotomy were then performed. The uterus was retrieved transvaginally, followed by laparoscopic vaginal cuff closure, hemostasis, and pelvic inspection. Finally, the trocars were removed and the skin closed. The robotic instruments used for this approach included the monopolar scissors, a bipolar Maryland grasper, a bipolar Johann grasper, and a needle driver.

### Sample size and statistical analysis

A sample size of 52 subjects was selected to provide robust estimates for conversion and adverse event rates. At least 45 subjects completing follow-up were needed to meet clinical acceptance criteria set at 10% for the primary performance endpoint, according to the literature screening on similar robotic systems performance, as discussed below. With one conversion in 45 patients, the 95% confidence upper limit is 10.1%. For safety, if 5 serious adverse events occur in 45 patients (11.1%), the upper 95% confidence limit is 22.0%, which remains within acceptable bounds. Descriptive statistics were used, with continuous variables summarized by the number of observations (N), mean, standard deviation (SD), and 95% confidence interval of the mean (95% CI). No statistical significance was tested given the single-arm design of the study.

## Results

A total of 52 patients were enrolled in the study and underwent total RAH using DEXTER between June 2024 and February 2025. The mean age was 43.0 ± 11.7 years, and the mean BMI was 24.5 ± 4.3 kg/m^2^. Most participants (82.7%) were classified as ASA II, while 17.3% were ASA I. Table 1 provides an overview of demographic data of the studied population as well as their indications for surgery. The most common indications for surgery were abnormal uterine bleeding (40.4%), fibroids (23.1%), and symptomatic adenomyosis (21.2%). Additional indications classified as “Other” (9.6%) in Table 1 included chronic pelvic pain, ovarian cyst with a history of breast cancer, a BRCA1 mutation, uterine cavity polyp, and lower urinary tract symptoms.

**Table 1:** Demographics and indications for surgery

Parameter (*N* = 52)	Value
Age (years)	
Mean ± SD (95% CI of mean)	43.0 ± 11.7 (39.8, 46.3)
BMI (kg/m^2^)	
Mean ± SD (95% CI of mean)	24.5 ± 4.3 (23.4, 25.7)
Uterine weight (g), *n* = 51*	
Mean ± SD (95% CI of mean)	151.1 ± 116.1 (118.4, 183.7)
ASA status (% (n/N))	
ASA I—a normal healthy subject	17.3% (9/52)
ASA II—a subject with mild systemic disease	82.7% (43/52)
ASA III—a subject with severe systemic disease	0.0% (0/52)
Indication for surgery (% (n/N))	
Abnormal uterine bleeding	40.4% (21/52)
Fibroids	23.1% (12/52)
Symptomatic adenomyosis	21.2% (11/52)
Pelvic pain or inflammation	19.2% (10/52)
Endometriosis	13.5% (7/52)
Gender dysphoria	11.5% (6/52)
Menstrual disorders	7.7% (4/52)
Persistence of human papillomavirus	5.8% (3/52)
Premalignant lesion of the endometrium and cervix	5.8% (3/52)
Cervical dysplasia	3.8% (2/52)
Other	9.6% (5/52)

^*^Data not recorded for one subject

Concomitant adnexal surgery was performed in all cases, with salpingectomy in 80.8%, salpingo-oophorectomy in 17.3%, and oophorectomy in 1.9% of subjects. The majority of adnexal procedures were bilateral (98.1%). All procedures were completed successfully using DEXTER without conversion to open or conventional laparoscopic surgery. The mean total skin-to-skin operative time was 121.9 ± 42.7 min, with a mean console time of 77.2 ± 29.4 min, and a mean docking time of 3.8 ± 1.3 min (Table 2). The mean estimated blood loss data, recorded for 51 of the 52 patients (one missing value), was 87.8 ± 93.8 mL. Uterine weight was likewise missing for one patient, resulting in a mean uterine weight of 151.1 ± 116.1 g for the 51 cases. A moderate positive correlation was observed between uterine weight and total skin-to-skin operative time (r = 0.459, *p* = 0.0007), indicating that larger uterine size was associated with longer operative times. In 11 cases, the procedure was completed using only 3 ports for the 3 robotic arms operated by the surgeon at the sterile console with assistance provided by a scrub nurse only for occasional endoscope cleaning or robotic instrument exchange. Of those cases, ten were total hysterectomies with salpingectomy, and one was a total hysterectomy with salpingo-oophorectomy, whereby the uterine weight of those patients ranged from 84 to 344 g. In the remaining cases, an additional assistant trocar was used as needed.

**Table 2:** Procedural details

Parameter (*N* = 52)	Value
Conversions to open surgery or laparoscopy (% (n/N))	0.0% (0/52)
Total operative time (min)	
Mean ± SD (95% CI of mean)	121.9 ± 42.7 (110.0, 133.8)
Console time (min)	
Mean ± SD (95% CI of mean)	77.2 ± 29.4 (69.0, 85.4)
Docking time (min)	
Mean ± SD (95% CI of mean)	3.8 ± 1.3 (3.4, 4.2)
Use of additional/assistant trocar sites (% (n/N))	
No	21.2% (11/52)
Yes	78.8% (41/52)
Estimated blood loss (mL), *n* = 51*	
Mean ± SD (95% CI of mean)	87.8 ± 93.8 (61.5, 114.2)
Intraoperative complications (% (n/N))	
No	100.0% (52/52)
Yes	0.0% (0/52)
Blood transfusions (% (n/N))	
No	100.0% (52/52)
Yes	0.0% (0/52)
Concomitant adnexal surgery (% (n/N))	
Salpingectomy	80.8% (42/52)
Salpingo-oophorectomy	17.3% (9/52)
Oophorectomy	1.9% (1/52)
Type of adnexal procedure (% (n/N))	
Unilateral	1.9% (1/52)
Bilateral	98.1% (51/52)

^*^Data not recorded for one subject

Initial adhesiolysis was performed in 22 patients, robotically in 20 cases and laparoscopically in 2. In these two cases, the surgeon chose the laparoscopic approach based on their preference due to right iliac fossa adhesions in one case, and history of peritonitis in another. In both instances, once adhesiolysis was completed, DEXTER was used to complete all remaining steps of the procedure. In all other cases, the entirety of the procedure, including initial adhesiolysis, total hysterectomy, and adnexal surgery, was performed fully robotically. In five patients (9.6%), vaginal morcellation was performed.

No patients required percutaneous drainage. Eight patients (15.4%) were discharged within 24 h, two of whom were discharged under an enhanced recovery after surgery (ERAS) protocol. One patient experienced a delayed discharge due to an event unrelated to the procedure. The mean time to discharge was 2.0 ± 0.9 days. Most patients (82.7%) did not receive opioids after discharge, and only 9.6% required antibiotics (Table 3).

**Table 3:** Postoperative outcomes

Parameter (*N* = 52)	Value
Reoperation prior to discharge (% (n/N))	0.0% (0/52)
Discharge	
Discharge within 24 h (% (n/N))	15.4% (8/52)
Delayed by events not related to the procedure (% (n/N))	1.9% (1/52)
Days to discharge (overall)	
Mean ± SD (95% CI of mean)	2.0 ± 0.9 (1.7, 2.2)
Need for percutaneous drainage (% (n/N))	0.0% (0/52)
Opioids prescription at discharge (% (n/N))	17.3% (9/52)
Antibiotics prescription at discharge (% (n/N))	9.6% (5/52)
Rehospitalization since discharge (% (n/N))	5.8% (3/52)
Reoperation since discharge (% (n/N))	1.9% (1/52)
Mortality (% (n/N))	0.0% (0/52)
Device-related adverse events (% (n/N))	0.0% (0/52)
Clavien-Dindo postoperative adverse events (% (n/N))	
Grade I	21.2% (11/52)
Bleeding, hematoma, postoperative subileus, abdominal pain with cough, infection, nausea and vomiting, gastroenteritis, discovery of endometrial adenocarcinoma, vaginal cuff dehiscence	
Grade II	25.0% (13/52)
Bleeding, mild anemia, wound infection, infected hematoma, genitourinary tract infection, abdominal pain with diarrhea and nausea, allergic reaction	
Grade IIIb	1.9% (1/52)
Bleeding from the vaginal cuff	
Grade IV-V	0% (0/52)

There were no reported adverse events related to DEXTER. Postoperative adverse events of Clavien–Dindo grade I occurred in 21.2% of subjects (11/52); grade II adverse events were observed in 25.0% of subjects (13/52) (Table 3). A single grade IIIb adverse event (1.9%) was reported, involving vaginal cuff bleeding due to suture dehiscence on postoperative day 13, requiring reoperation to repair the sutures. At the 42-day follow-up visit, the cervical stump was confirmed to have healed, and the event had resolved without any sequalae. No grade IV or V adverse events occurred.

Two other patients were rehospitalized: one for fever and elevated infection markers, which were treated successfully with intravenous antibiotics, without sequalae, and the other for postoperative vaginal bleeding, which resolved spontaneously, with no further adverse events reported through the 42-day follow-up appointment.

## Discussion

In our multicenter study, all procedures were successfully completed without conversion to open or conventional laparoscopic surgery, fulfilling the primary performance endpoint and demonstrating that DEXTER performed consistently in RAH across various clinical settings, with outcomes comparable to other robotic systems. No conversions of any kind occurred during the study, while conversion rates reported in the literature on comparable devices range from 0–3.1% for open surgery [16, 17] and up to 0.2% for laparoscopy [18].

Only one major adverse event (Clavien–Dindo grade IIIb) occurred, resulting in a major adverse event rate (1.9%) well within the acceptable threshold and consistent with published data (0–7.1%) [19, 20]. Importantly, this event was assessed as not device-related. These findings support the safety of DEXTER across different clinical settings when used as intended for RAH. The overall complication rate of 37.3%, consisting primarily of minor adverse events (Clavien–Dindo grade I–II), also falls within the range reported in previous studies [21–23], and reflects comprehensive and rigorous documentation practices inherent to this prospective, 100% monitored study design. Such low-grade events are often underreported in retrospective studies, potentially contributing to lower adverse event rates in the reported data, therefore making overall adverse events an unreliable metric for comparing the safety of new robotic systems to the literature on older systems. Unfortunately, there is currently a lack of publications of large-scale, prospective registry data for robotic-assisted hysterectomy, limiting the availability of directly comparable benchmarks. Consequently, overall complication rates, particularly for Clavien–Dindo grade I–II events, should be interpreted in light of the study design and reporting methodology. The mean intraoperative blood loss of 87.8 ± 93.8 mL was comparable to values reported in the literature for other robotic platforms (5–250 mL) [16, 24, 25], and the mean length of hospitalization was similarly within the published range (0.9–7.2 days) [26, 27].

Intraoperative performance metrics support the operational efficiency of DEXTER: mean total skin-to-skin operative time was well in range for those reported for benign hysterectomy (73.9–241.8 min) [17, 28, 29] with all critical steps of total hysterectomy and concomitant procedures completed robotically. Mean docking time was below 4 min, indicating an easy and quick setup of the robotic system, also possible due to its open architecture, as familiar laparoscopic tower equipment and workflows were employed.

In 21.2% of cases, with small- to moderate-sized uteri, procedures were completed using only three robotic ports without an additional assistant trocar. This streamlined approach may contribute to reduced postoperative pain and improved cosmetic outcomes, factors known to influence patient satisfaction in surgery [30]. This technique was facilitated by the sterile console setup, allowing the surgeon direct patient access without breaking sterility.

The eight patients (15.4%) discharged within 24 h postoperatively suggest the system’s potential for integration into outpatient care pathways. However, in most countries, reimbursement frameworks favor inpatient care, and robotic-assisted gynecologic surgery is not reimbursed at higher rates than conventional approaches, regardless of setting. While ambulatory surgery is increasingly encouraged, current reimbursement structures do not adequately support the higher costs associated with traditional robotic systems, limiting their use in cost-sensitive environments such as ambulatory surgery centers.

DEXTER addresses these barriers through its simple setup, compatibility with standard laparoscopic equipment, and minimal infrastructure requirements, reducing investment burdens while allowing efficient workflows. This system demonstrated adaptability across both larger university and smaller community centers, facilitated by its compact, mobile design that accommodates even limited OR space. Moreover, by enabling quick transitions between laparoscopic and robotic modalities, DEXTER offers a flexible solution for diverse surgical environments. Its safety and performance across a range of hospital settings suggest it may be a cost-effective alternative to conventional robotic platforms and may support broader adoption of minimally invasive hysterectomy, particularly in systems navigating financial constraints and policy shifts toward ambulatory care. Its modular, mobile design, and compatibility with existing OR equipment reduce both logistical and financial barriers to adoption.

Limitations of this study include its single-arm design and lack of a direct comparator. In addition, participating surgeons had varying levels of prior robotic experience, and some were still early in their learning curve with DEXTER, which potentially influenced the surgical outcomes. While the overall operative performance was consistent across centers, variability in operative times was observed and may partially reflect differences in surgeon experience. However, the 100% procedural success rate and absence of conversions across this heterogeneous group suggest that DEXTER can be safely and effectively adopted by surgeons at different stages of robotic proficiency. Future studies with larger sample sizes will allow formal stratification of learning curve effects. In addition, while the 42-day follow-up period was sufficient to confirm vaginal cuff healing and immediate postoperative complications, it does not allow for assessment of longer term sequelae, such as late-onset pelvic floor dysfunction, delayed complications, or longer term quality-of-life outcomes. Longer term data would further validate these early findings. A larger cohort would also improve detection of rarer adverse events.

## Conclusion

This study demonstrates that the DEXTER Robotic Surgery System is safe, effective, and efficient for benign hysterectomy with adnexal surgery across a range of clinical environments. Its design and performance characteristics position it as a promising platform to broaden access to robotic-assisted surgery, especially in outpatient settings, where traditional systems may not be accessible. Future studies should further investigate long-term outcomes, learning curves, and economic impact across diverse healthcare systems.

## Data Availability

The datasets generated during and/or analyzed during the current study are available from the corresponding author on reasonable request.
